# Predictive factors for docking site procedure in bone transport for large lower extremity segmental defects

**DOI:** 10.1186/s12891-023-06593-6

**Published:** 2023-06-17

**Authors:** T. Omar Pacha, G. Aktas, T. Graulich, T. Stübig, J. D. Clausen, E. Liodakis, M. Omar, S. Sehmisch, P. Mommsen

**Affiliations:** Trauma Department, Hannover Medical School (MHH), Lower Saxony, Germany

## Abstract

**Background:**

Segmental bone transport is a common technique for treating large segmental bone defects. However, a docking site procedure is often necessary in segmental bone transport. To date, no prognostic factors for the need of docking site procedure have been reported. Thus, the decision is often made at random, based on the surgeon’s subjective judgment and experience. The aim of this study was to identify prognostic factors for the need of docking site operation.

**Methods:**

Patients with segmental bone transport in lower extremity bone defects were included regardless of age, aetiology, and defect size. We excluded patients undergoing treatments that were not yet completed, and those who discontinued therapy by any reason. The need for docking site operation was modelled with logistical and linear regression as well as univariate analysis of variances (ANOVA). Receiver operating characteristics (ROC) curve analysis was also performed.

**Results:**

Twenty-seven patients from age 12 to 74 years (mean age: 39.07 ± 18.20 years) were included. The mean defect size was 76.39 ± 41.10 mm. The duration of transport (days) showed a significant influence (p = 0.049, 95%CI: 1.00–1.02) on the need for docking site operation. No other significant influences were detected.

**Conclusion:**

A link between the duration of transport and the need for docking site operation was detected. Our data showed that if a threshold of about 188 days is exceeded, docking surgery should be considered.

**Supplementary Information:**

The online version contains supplementary material available at 10.1186/s12891-023-06593-6.

## Introduction

Major segmental defects often occur in patients with severe injury but also as consequence of tumour and infection [1]. Bone defects of the lower extremity that are > 5 cm in diameter are considered critical [2, 3]. When this threshold is reached, complex surgical procedures like vascularized bone transplantation, segmental bone transport, or Masquelet technique are required [2–4].

Although bone transport is associated with a high psychological, economic, and social burden [2, 3], it is a common technique for treating large segmental bone defects[5]. A docking site procedure is often necessary in segmental bone transport [2, 6]. Garcia et al. [7] reported a histological analysis in nine adult sheep showing clear differences in ossification between the regenerate and docking site. In contrast to intramembranous ossification in the regenerate, docking site predominantly showed endochondral ossification [7]. Thus far, to our knowledge, no prognostic factors for the need of docking site procedure have been reported. Therefore, the decision between spontaneous healing of the docking site versus surgical intervention is more often made by trial and error, as well as the applied technique to ensure the docking site connection, which is often chosen as the personal best known surgical technique, regardless any objective data recommending plate osteosynthesis, intramedullary nailing or engagement of ring fixators. [6, 8–10]. In our department, when it comes to docking site procedure, the authors usually tend to perform plate osteosynthesis, thus we have good results in the last years and contraindications like infection or soft tissue problems are ruled out. Recently we establish the usage of personalized 3D-printed titanium plates to address the extreme individualized circumstances of the anatomy after segmental bone transport.

Apart from the very time consuming nature of the segmental bone transport procedure [2, 11], further time is lost when waiting for spontaneous healing of the docking site and periods of further compression and application of additional treatments like pulsed low-intensity ultrasound [12]. Despite these efforts, the refusal of surgical intervention often turns out to be wrong, resulting in a non-union of the docking site. However, we retrospectively analysed 27 patients who underwent a segmental bone transport for lower extremity bone defects between January 2013 and October 2021 in our department. We aimed to identify prognostic factors for the need for docking site operation (transport speed, duration of segmental bone transport and defect length).We assumed that a specific threshold of transport duration was predictable for docking site surgery to ensure sufficient osseointegration of the docking site as spontaneous healing was not to be expected.

## Methods

### Inclusion and exclusion criteria

In this retrospective analysis, we enrolled all patients treated in our department between January 2013 and October 2022 for lower extremity bone defects by segmental bone transport. Patients of all ages and both sexes who required segmental bone transport procedure, regardless of aetiology and defect size, were included.

We excluded patients whose treatment was not yet completed and those who discontinued treatment for any reason.

### Definitions

The defect size measured in millimeters (mm) was determined postoperatively at the beginning of bone transport. The duration of transport was defined as the time period between transportation start and the last day of transport or the date of docking procedure. The mean transportation speed was calculated as the ratio of transport duration and defect size in mm/day. All radiological analyses and measurements were carried out by the same investigator using the program Visage Version 7.1 (Visage Imaging, Berlin, Germany).

### Clinical parameters and surgical procedures

Baseline values (age, sex, comorbidities, fracture type in case of traumatic bone defects) as well as type of injury, reason of bone defect, and location of bone defect were obtained. For segmental bone transport, four different surgical approaches were applied. Besides external transport devices (ring fixator, monorail fixator) the only internal transport system we used was the intramedullary PRECICE bone transport nail (NuVasive, San Diego, CA, USA). Our department uses the classic Ilizarov ring fixator; and LRS monorail fixator (Orthofix, Lewisville, TX, USA) as external transport systems. The fourth group resembles a combining of a classic external segmental transport by the LRS monorail system and the first step of the Masquelet technique, which literature recently reported as piston technique [13]. Therefore, expecting an infection on the fracture site in situations of non-healing bones, resection of dead and infected bone, and implanting bone cement spacer is often necessary to get an infection-free defect area. Removing the spacer 6–8 weeks after and initiating segmental bone transport is very similar to the first step of the Masquelet technique, but instead of cancellous filling (Masquelet technique), the second step (cancellous filling) was replaced by segmental transport [13].

### Statistical analysis

In the present explorative study, the influence of potential predictive factors on the need for docking site operation was modelled with logistical and linear regression as well as univariate analysis of variances (ANOVA). Defining the match of sensitivity and specificity, predictive values of the different parameters were estimated using receiver operating characteristic (ROC) curve analysis and area under the curve (AUC). Optimal cut-off points for each predictive factor were determined by the maximized Youden’s index. Results are presented as standard error of mean (SEM), standard deviation (SD), variances, odds ratio (OR), and two-sided 95% confidence intervals (95%CI). Graphical tools like scatter plot, histogram, and box plots were employed. The level of statistical significance was considered at p < 0.05. Statistical analysis was performed using SPSS computer software (SPSS 28, IBM, Armonk, NY, USA). Only fully anonymized data were evaluated.

#### Ethical approval

, *informed consent, and funding*.

This research was performed in accordance with the Declaration of Helsinki and has been approved by the local Hannover Medical School Research Ethics Committee. No concerns have been raised (10517_BO_K_2022). Written informed consent was obtained from all patients. In case of minor participants, the informed consent by a parent and/or legal guardian was obtained.

## Results

### Clinical data

In all, 27 patients (21 male and 6 female) aged between 12 and 74 years (mean age: 39.07 ± 18.20 years) were included in the study (Table 1). Of these, 24 patients suffered a trauma-associated bone defect, whereas two bone defects were due to tumour resection. In one patient, the initial cause of the resulting bone size defect was not clear because of a lack of documentation in childhood. In all, we found 18 pseudoarthrosis of different aetiologies: 15 pseudoarthrosis were atrophic and three were hypertrophic. An infection was detected in 22 of 27 patients.

The mean defect size was 76.39 ± 41.10 mm, and the mean transport duration was 224.63 ± 128.06 days with a mean transportation speed of 0.39 ± 0.16 mm/day (Table 1). We included 12 femoral and 15 tibial defects.

**Fig. 1:**
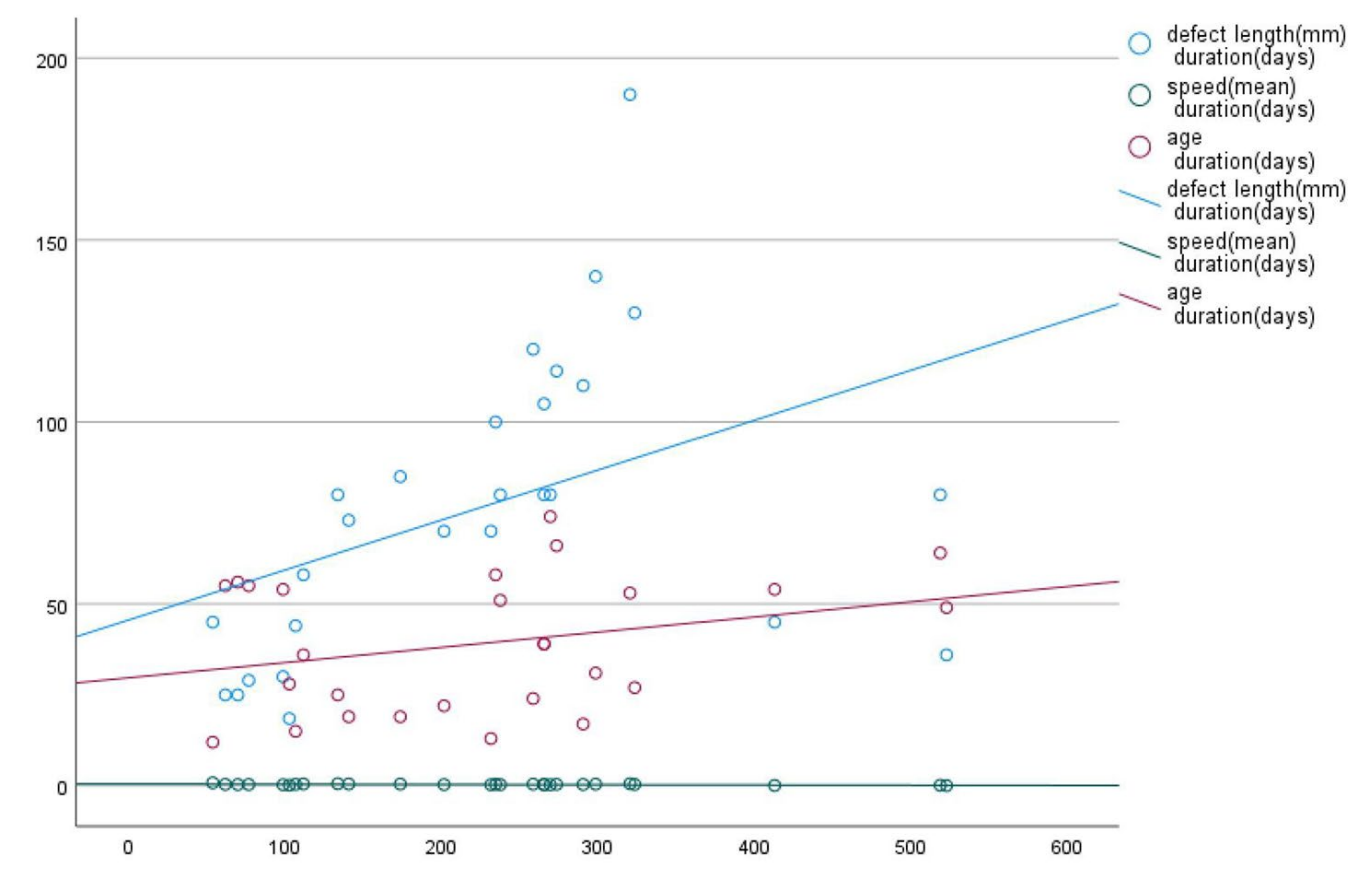
Correlation of transport duration with defect length, age, and transport speed

The distribution of patients according to the aforementioned surgical approaches were ring fixator (n = 5), mono rail fixator (n = 6), PRECICE nail (n = 8), mono rail fixator in terms of the piston technique (n = 8). A docking site procedure was performed in 18 of 27 patients. Figure 1 shows the correlation of transport duration with defect length, age, and transport speed.

### Predicting factors for docking site procedure

**Fig. 2a:**
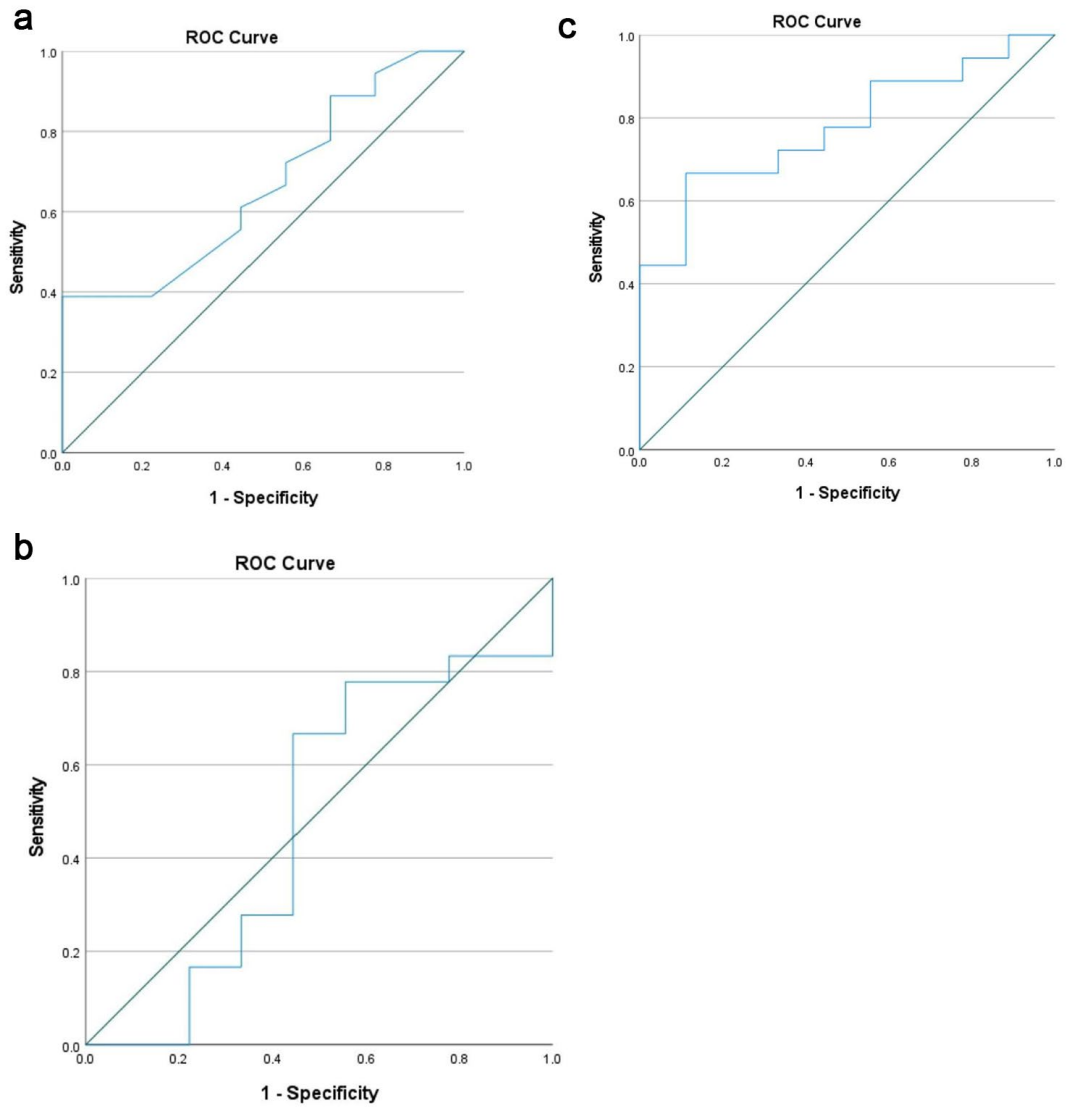
ROC curve for defect size: Cut-off value 51.5 mm shows a sensitivity of 0.72 and a specificity of 0.44. AUC = 0.66. **b** ROC curve for mean transport speed: Cut-off value is 0.32 mm/d with a sensitivity of 0.78 and a specificity of 0.44. AUC = 0.48. **c** ROC curve for duration of transport: Cut-off level of 188 days with a sensitivity of 0.72 and specificity of 0.67 with an AUC of 0.78

The results of the logistical regression analysis of docking site procedure-influencing factors such as defect length (mm), transport speed (mean), transport duration (days), age and sex are shown in Table 2. Only the duration of transport (days) showed a significant influence (p = 0.049, 95%CI: 1.00–1.02) on the need for docking site operation. The results of the ROC analysis including AUC and odds ratio for predicting necessary docking site operation with optimal cut-off points for defect size, transportation speed, and duration are shown in Table 3. For the defect size (cut-off value: 51.5 mm) a sensitivity of 0.72 and specificity of 0.44 was calculated with an AUC of 0.66 (Fig. 2a). In size defect, the odds ratio was 2.08 (95% CI: 0.39–11.06). Concerning the mean transportation speed, the optimal cut-off point was 0.32 mm/day predicting the need for a docking site procedure with a sensitivity of 0.78 and a specificity of 0.44 (AUC: 0.48; OR: 2.80 [95% CI: 0.50–15.66]) as shown in Fig. 2b. With a cut-off point of 188 days (0.72 sensitivity, 0.67 specificity, AUC = 0.78, and OR = 5.20 [95% CI: 0.92–29.26]), the transport duration seemed the most reliable predicting factor for the need of docking site operation (Fig. 2c).

**Fig. 3:**
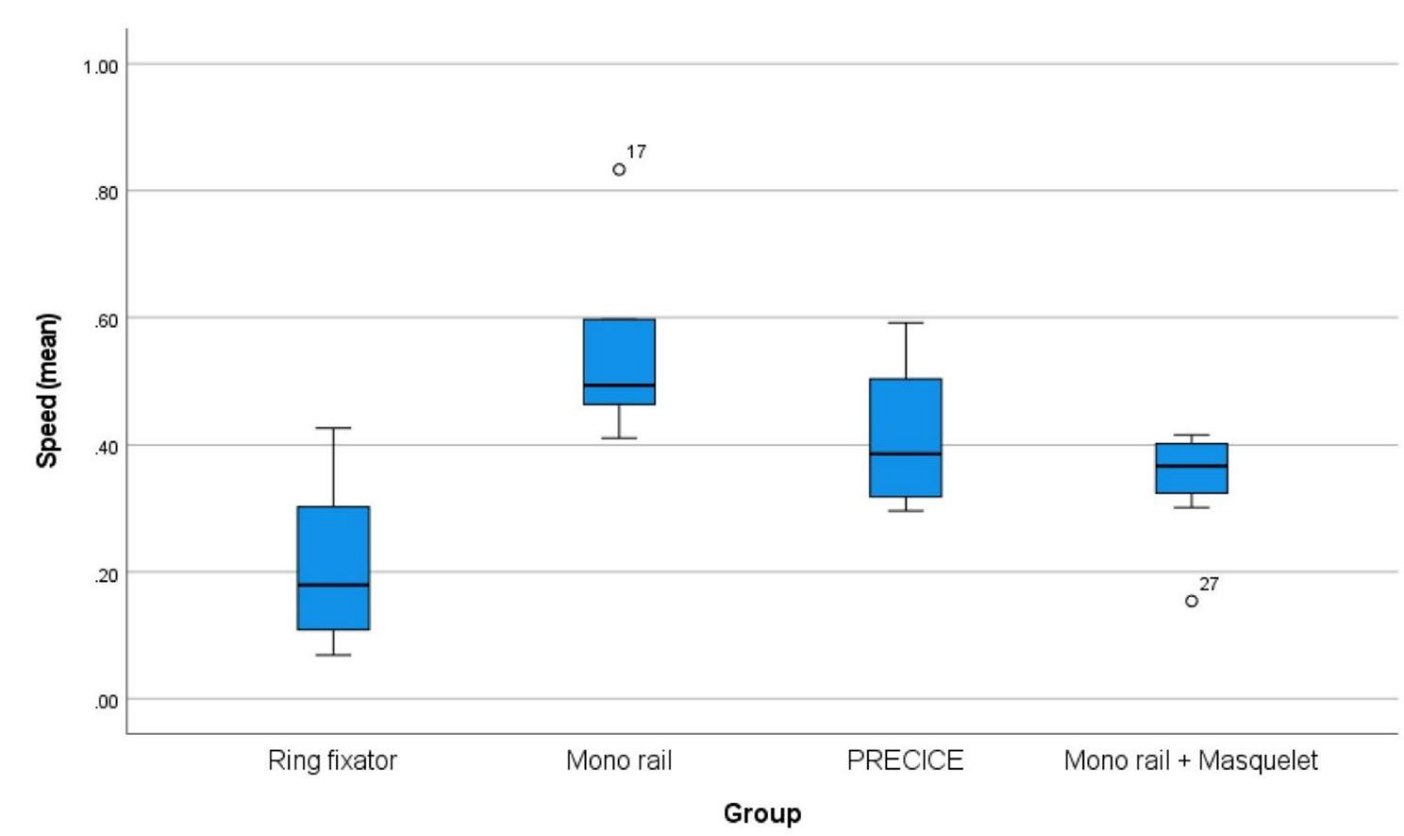
Difference in transport speed in all fourgroups

To avoid overlooking of relevant factors for daily use in segmental bone transport, the influence of age on transport speed was also calculated. A linear regression analysis was performed. While a moderate correlation (R = 0.41) could be reported, a significant dependence was found (p = 0.034, 95%CI: -0.007 to 0.00). Comparing the four treatment groups for transport speed, ANOVA showed a significant difference (p = 0.001) across all groups (Fig. 3). The biggest difference in transport speed was between the Ilizarov and LRS nono rail fixator. The mono rail fixator was 0.33 ± 0.10 mm/day faster than transport by Ilizarov ring fixator (p = 0.005, 95%CI: -0.54 to 0.13).

**Table 1:** Baseline characteristics of the study population for age, defect size (mm), duration of transport (days), and transport speed (mean). Standard statistical values are presented as mean values, standard error of mean (SEM), median, standard deviation (SD), variance, and minimum and maximum

	Age	Defect length (mm)	Duration (days)	Speed (mean)
Mean	39.07	76.39	224.63	0.39
Median	39.00	80.00	235.00	0.39
Standard deviation	18.55	41.10	128.10	0.16
Variance	344.00	1688.55	16398.10	0.03
Minimum	12.00	18.50	54.00	0.07
Maximum	74.00	190.00	523.00	0.83

**Table 2:** Logistical regression analysis of docking procedure-influencing factors such as defect length (mm), transport speed (mean), transport duration (days), age and sex.Standard error of mean (SEM), level of significance, odds ratio (Exp(B), and confidential interval (95%CI)

SEM	p	Exp(B)	95%CI		
			lower	upper	
Defect Length (mm)	0.01	0.13	1.02	1.00	1.05
Speed (mean)	2.70	0.44	0.12	0.00	24.11
Duration (days)	0.01	0.049	1.01	1.00	1.02
Age	0.02	0.78	1.01	0.96	1.05
Sex	0.98	1.00	1.00	0.15	6.85

**Table 3:** Association between defect size, transportation speed, transport duration, and docking site procedure

Parameter	AUC	95%CI	Cut-off*	Sensitivity	Specificity	p**	OR**
Defect size (mm)	0.66	0.45–0.88	51.5	0.72	0.44	0.134	2.08
Transport speed (mm/day)	0.48	0.23–0.74	0.32	0.78	0.44	0.435	2.80
Transport duration (days)	0.78	0.60–0.95	188	0.72	0.67	0.049	5.20

*Optimum cut-off as indicated by the maximized Youden’s index and ROC curve

**p value/OR as indicated by multivariate logistic regression

The boxplot shows a significant difference in treatment groups (p = 0.001). The black bar represents the median. Blue bars represent the interquartile range between the first and third quartile. Whiskers represent data within 1.5x of the interquartile range. Data falling outside of the 1.5x interquartile range but within the 3x interquartile range are plotted as outliers with a circle.

## Discussion

In the present study, 27 patients with different types of segmental bone transport in lower extremity bone defects were assessed in regards to predicting factors for the need of docking site procedure. The most reliable predicting factor was transport duration with a valid cut-off value higher than 188 days of transport. To the authors’ knowledge, such a discriminating factor has not yet been reported in literature. In the present study group, age, sex, comorbidities, transport speed, and defect size had no significant influence on the prediction of necessary docking site operation.

The treatment of larger bone defects is often followed by a long period of non-surgical docking attempts to avoid unnecessary surgery procedures in this very vulnerable area. Beside compression, increasing limb bearing, and application of external physical factors such as pulsed low-intensity ultrasound, no other high-impact concepts have yet been reported. Regarding everyday practice in the follow-up treatment for this highly demanding injury and the significant psychological, social, and economic burden on patients, a predictive cut-off value for good decision-making concerning docking site procedures seems reasonable. In this study, comorbidities had no impact on the need of docking site procedure. However, this result must be regarded with caution, because the number of cases for the high variability in comorbidities is small with an even smaller number of detected comorbidities (DM 2, renal insufficiency) and is therefore prone to bias. Apart from these mentioned restrictions, there are other limitations that can be identified. Given the considerably varying situations in which such defects can occur, the combination of fracture degree, type of long bone, comorbidities, and applied device can differ greatly. As a consequence, the four subgroups are not exactly matched concerning the number of cases with an additional imbalance in the distribution of sex and the frequency of segmental bone defects after third-degree open fractures.

This study reports a difference in speed of transportation regarding the applied transport device. The differences concerning the transportation speed, depending on the applied device maybe biased and femoral fractures and tibial fractures were also included as well, thus the usual transport speed for both location differs in millimeters per day. Taking the recommended difference in transport speed of those two areas (tibial 0.5 mm/day and femoral 1 mm/day) into account, an undetected bias is possible.

The only parameter we found truly significant in predicting the need of docking surgery was the duration of transport (p = 0.049). A cut-off value of 188 days of transport was detected (sensitivity: 72%, specificity: 67%, AUC: 0.78) that displayed good model quality.

Despite the listed limitations, the question of docking procedure is important when treating patients with large segmental bone defect, regardless of the device used for bone reconstruction. Thus, comorbidities like DM 2, peripheral vascular disease, nicotine abuse, and other bone nutrition-affecting diseases do not influence the decision of docking site surgery in the reported study population as no significant influence was detected. However, these results need to be validated in a larger study groups. Our data show that for a transport duration under 188 days, regardless of transport speed, age, bone type, and degree of fracture, a non-surgical attempt at healing and a satisfying union in the docking site may be successful. After exceeding the limit of 188 days, a docking procedure should be considered in the early stage of treatment of such defects.

## Conclusion

In this study, a link between the need for docking site procedure and bone transport duration was detected. Our data suggest that if a threshold of about 188 days is exceeded, docking surgery should be considered. To validate this proposed threshold, further studies have to be conducted with a higher number of patients and more balanced subgroups.

## Electronic supplementary material

Below is the link to the electronic supplementary material.

Supplementary Material 1

## Data Availability

All data generated or analyzed during this study are included in this published article and its supplementary files.
